# Modeling and Assessment of Precise Time Transfer by Using BeiDou Navigation Satellite System Triple-Frequency Signals

**DOI:** 10.3390/s18041017

**Published:** 2018-03-29

**Authors:** Rui Tu, Pengfei Zhang, Rui Zhang, Jinhai Liu, Xiaochun Lu

**Affiliations:** 1 National Time Service Center, Chinese Academy of Sciences, Shu Yuan Road, Xi’an 710600, China; zpf.333@163.com (P.Z.); zhangruiwkk@163.com (R.Z.); jinhailiu15@163.com (J.L.); luxc@ntsc.ac.cn (X.L.); 2 Key Laboratory of Precision Navigation and Timing Technology, Chinese Academy of Sciences, Shu Yuan Road, Xi’an 710600, China; 3 University of Chinese Academy of Sciences, Yu Quan Road, Beijing 100049, China; 4 State Key Laboratory of Geo-Information Engineering, Yan Ta Road, Xi’an 710054, China

**Keywords:** BeiDou, triple-frequency signals, time transfer, precise point positioning

## Abstract

This study proposes two models for precise time transfer using the BeiDou Navigation Satellite System triple-frequency signals: ionosphere-free (IF) combined precise point positioning (PPP) model with two dual-frequency combinations (IF-PPP1) and ionosphere-free combined PPP model with a single triple-frequency combination (IF-PPP2). A dataset with a short baseline (with a common external time frequency) and a long baseline are used for performance assessments. The results show that IF-PPP1 and IF-PPP2 models can both be used for precise time transfer using BeiDou Navigation Satellite System (BDS) triple-frequency signals, and the accuracy and stability of time transfer is the same in both cases, except for a constant system bias caused by the hardware delay of different frequencies, which can be removed by the parameter estimation and prediction with long time datasets or by a priori calibration.

## 1. Introduction

Precise time is critical for military strikes, as well as for national infrastructure facilities, such as electric power supply, banks, and communication networks [1]. Precise time transfer is essential for supporting and guaranteeing a precise time service.

At present, time transfer can be performed using BPM/BPL (ground-based radio stations that broadcast short-wave and long-wave time signals, respectively), two-way satellite time and frequency transfer (TWSTFT), optical fiber (OP), and global navigation satellite system (GNSS) [1]. Among these approaches, GNSS time transfer is preferable because of its portability and low cost [2].

Most timing laboratories widely use common-view (CV) and all-view (AV) techniques for GNSS time transfer. In CV, signals from the same satellites are collected to remove common observation errors at ground stations. However, the CV condition for the same satellites is greatly limited by the distance of the ground stations, and the CV time transfer accuracy is only ~0.3 ns owing to the precision of the related pseudo-range measurement [3,4]. In AV, the GNSS system time is used for precise time transfer, and the CV condition is not necessary. AV enables precise time transfer at any time and at any station [5]. Carrier phase (CP) precise point positioning (PPP) is a typical AV technique with subnanosecond accuracy that is used for carrier phase observation.

The BeiDou Navigation Satellite System (BDS) has been providing a local positioning, navigation, and timing (PNT) service since the end of 2012, and it will be expanded to a global PNT service at the end of 2020 [6]. Currently, the accuracy of the BDS one- and two-way timing services can reach 20–100 ns and 10–20 ns, respectively. BDS currently uses only low-precision pseudo-range measurement for both timing services, and it does not yet fully use high-precision CP measurements. Separately, most studies have investigated dual-frequency signals for precise time transfer; however, the triple-frequency signals used in BDS have not been investigated in detail [7,8,9]. In particular, the data solution model needs to be studied when using triple-frequency signals for precise time transfer.

Therefore, the main contribution of this study is that it proposes two models for precise time transfer by using BDS triple-frequency signals: ionosphere-free (IF) combined precise point positioning (PPP) model with two dual-frequency combinations (IF-PPP1) and ionosphere-free combined PPP model with a single triple-frequency combination (IF-PPP2). Secondly, it evaluates the performance of these models for BDS time transfer. The results shown that IF-PPP1 and IF-PPP2 models can both be used for precise time transfer using BDS triple-frequency signals, and the accuracy and stability of time transfer is the same in both cases. The coordinates and troposphere delay estimates also have the same precision. In addition, the constant system bias caused by the hardware delay of different frequencies is stable and can be removed by the parameter estimation and prediction with long time datasets or by a priori calibration. These models and validation results will be important for the BDS precise time transfer.

This manuscript is arranged as follows. First, a model for time transfer using dual-frequency signals is introduced. Next, two different models for time transfer using triple-frequency signals are presented. These models are validated using different experimental data. Finally, some conclusions and discussions are presented.

## 2. Dual-Frequency Time Transfer Model for BDS

This section presents mathematical models, specifically, the observation model and stochastic model, for precise time transfer using dual-frequency ionosphere-free (IF) combination observations (IF-PPP0) in BDS.

### 2.1. Observation Model

Usually, precise time transfer using the CP approach uses the PPP model. The mathematical model for dual-frequency observations using the IF combination can be expressed as follows [10,11].

(1) $$P_{12}=\alpha_{12}P_{1}+\beta_{12}P_{2}=\rho +c\cdot dt_{12}+d_{trop}+M_{P}+\varepsilon_{P_{12}}$$

(2) $$\phi_{12}=\alpha_{12}\phi_{1}+\beta_{12}\phi_{2}=\rho +c\cdot dt_{12}+d_{trop}+M_{\phi }+N_{12}+\varepsilon_{\phi_{12}}$$

where P and ϕ are the pseudo-range and CP measurements, respectively. The superscripts 1 and 2 are the index of the frequency; $\alpha_{12}=\frac{f_{1}^{2}}{f_{1}^{2}-f_{2}^{2}}$ and $\beta_{12}=-\frac{f_{2}^{2}}{f_{1}^{2}-f_{2}^{2}}$ are the coefficients of the IF combination; f is the frequency; ρ is the geometric distance from the satellite to the receiver; c is the velocity of light; ${\mathrm{dt}}_{12}$ is the receiver clock error by the $P_{1}$ and $P_{2}$ ionosphere-free combination; $d_{\mathrm{trop}}$ is the tropospheric delay; M represents other errors related to the solid tide, ocean tide, Earth’s rotation, relativity, phase center offset, phase wind up, and multipath; N is the ambiguity; and ε is the measurement noise.

### 2.2. Stochastic Model

Usually, the satellite elevation, signal-to-noise ratio, or a combination of both can be used to determine the stochastic model [12,13,14]. In this study, we use an elevation-dependent weighting scheme with a sine-mapping function. Under the assumptions of uncorrelated observations and the same a priori noise ($\sigma_{1}=\sigma_{2}=\sigma_{0}$) at all frequencies, the variance–covariance matrix of the original uncombined observations can be expressed as

(3) $$\sum UC=\left[\begin{array}{cc} \sigma_{1}^{2} & 0\\ 0 & \sigma_{2}^{2} \end{array}\right]=\sigma_{0}^{2}\left[\begin{array}{cc} 1 & 0\\ 0 & 1 \end{array}\right]$$

where $\sigma_{0}=a/\sin\left(E\right)$, a is a constant generally set to be 0.002–0.003 m for the carrier phase and 0.2–2.0 m for the code observations, and E is the satellite elevation angle (unit: rad).

By using the error propagation law, the covariance matrices for the IF-PPP0 models can be expressed as

(4) $$\sum IF-PPP_{0}=\sigma_{0}^{2}\left[\alpha_{12}^{2}+\beta_{12}^{2}\right]$$

## 3. Triple-Frequency Time Transfer model for BDS

This section presents two different time transfer models for BDS triple-frequency signals, namely, IF-PPP1 and IF-PPP2, as defined above.

### 3.1. IF-PPP1: Ionosphere-Free Model with Two Dual-Frequency Combinations

By using Equations (1) and (2), we can form a dual-frequency ionosphere-free combination for the triple-frequency signals B1, B2, and B3. Considering that the noise amplification factor of the B2/B3 combination is considerably larger than that of the B1/B2 and B1/B3 combinations, we only used the B1/B2 and B1/B3 combinations in this model [15].

If the receiver hardware delay has a common bias for the dual-frequency IF-PPP (using only B1/B2 or B1/B3), this bias will be absorbed by the receiver clock parameter; therefore, we do not need to consider it. However, the situation is different for the triple-frequency PPP once we use two or more IF combinations (B1/B2 and B1/B3). Because the receiver code biases are different for each IF combination, they cannot be compensated for by the receiver clock anymore. To account for this, separate clock parameters should be estimated for each IF combination. Alternatively, an inter frequency bias (IFB) parameter can be estimated in addition to the receiver clock bias. Here, we introduce an IFB parameter for the B1/B3 code combination. The receiver IFB is considered common to all satellites. Then, the linearized observation equations can be written as follows:

(5) $$P_{12}=\alpha_{12}P_{1}+\beta_{12}P_{2}=\rho +c\cdot dt_{12}+d_{trop}+M_{P}+\varepsilon_{P_{12}}$$

(6) $$P_{13}=\alpha_{13}P_{1}+\beta_{1n}P_{3}=\rho +c\cdot dt_{12}+IFB_{13}+DCB_{PPP1}^{S}+d_{trop}+M_{P}+\varepsilon_{P_{13}}$$

(7) $$\phi_{12}=\alpha_{12}\phi_{1}+\beta_{12}\phi_{2}=\rho +c\cdot dt_{12}+d_{trop}+M_{\phi }+N_{12}+\varepsilon_{\phi_{12}}$$

(8) $$\phi_{13}=\alpha_{13}\phi_{1}+\beta_{13}\phi_{3}=\rho +c\cdot dt_{12}+d_{trop}+M_{\phi }+N_{13}+\varepsilon_{\phi_{13}}$$

where IFB is the receiver interfrequency bias, ${\mathrm{dt}}_{12}$ is the ionosphere-free clock based on the P1 and P2 code IF combination, $\alpha_{13}=\frac{f_{1}^{2}}{f_{1}^{2}-f_{3}^{2}}$, and $\beta_{13}=-\frac{f_{3}^{2}}{f_{1}^{2}-f_{3}^{2}}$. Here, the differential code bias (DCB) is defined as ${\mathrm{DCB}}_{\mathrm{PPP}1}^{S}=\beta_{12}\cdot {\mathrm{DCB}}_{12}^{S}-\beta_{13}\cdot {\mathrm{DCB}}_{13}^{S}$.

In addition, the stochastic model can be expressed as follows:

(9) $$\sum IF-PPP_{1}=\sigma_{0}^{2}\left[\begin{array}{cc} \alpha_{12}^{2}+\beta_{12}^{2} & \alpha_{12}\alpha_{13}\\ \alpha_{12}\alpha_{13} & \alpha_{13}^{2}+\beta_{13}^{2} \end{array}\right]$$

### 3.2. IF-PPP2: Ionosphere-Free Model with a Single Triple-Frequency Combination

Unlike the IF-PPP1 model that forms two dual-frequency IF combinations (B1/B2 and B1/B3), the IF-PPP2 model directly combines the triple-frequency measurements within one combination (B1/B2/B3) as follows.

(10) $$P_{123}=e_{1}P_{1}+e_{2}P_{2}+e_{3}P_{3}=\rho +c\cdot \left(dt_{12}+IFB_{123}\right)+DCB_{PPP2}^{S}+d_{trop}+M_{P}+\varepsilon_{P_{123}}$$

(11) $$\phi_{123}=e_{1}\phi_{1}+e_{2}\phi_{2}+e_{3}\phi_{3}=\rho +c\cdot \left(dt_{12}+IFB_{123}\right)+d_{trop}+M_{\phi }+N_{123}+\varepsilon_{\phi_{123}}$$

Because all three pseudo-ranges are combined within the same code observation equation, the receiver hardware delay biases all the ranges by a constant (${\mathrm{IFB}}_{123}$) that will eventually be absorbed by the receiver clock parameter. Thus, we do not have to consider it for the parameter estimation. Here, ${\mathrm{DCB}}_{\mathrm{PPP}2}^{S}=(\beta_{12}-e_{2})\cdot {\mathrm{DCB}}_{12}^{S}-e_{3}\cdot {\mathrm{DCB}}_{13}^{S}$ and $\left[\begin{array}{ccc} e_{1} & e_{2} & e_{3} \end{array}\right]=\left[\begin{array}{ccc} 2.566 & -1.229 & -0.337 \end{array}\right]$.

In addition, the stochastic model can be expressed as follows:

(12) $$\sum IF-PPP_{2}=\sigma_{0}^{2}\left(e_{1}^{2}+e_{2}^{2}+e_{3}^{2}\right)$$

### 3.3. Conclusions of PPP Models for Precise Time Transfer

Table 1 summarizes the major characteristics of the precise time transfer models using triple-frequency BDS signals, including the observation used (Obs.), combination coefficients ($\begin{array}{ccc} e_{1} & e_{2} & e_{3} \end{array}$), noise amplification factor (Noise), satellite DCB corrections (Sat. DCB), and receiver DCB process approach (Rec. DCB).

For the IF-based PPP models, two highly correlated dual-frequency IF combinations (B1/B2 and B1/B3) are formed in the IF-PPP1 model, whereas the triple-frequency signals are combined within one optimal IF combination (B1/B2/B3) in the IF-PPP2 model. Note that the IF-PPP1 model is more flexible than the IF-PPP2 model in the absence of a particular frequency. A comparison of the combination coefficients of the IF-PPP1 and IF-PPP2 models shows that the IF-PPP2 model is more like the IF-PPP0 model owing to the lower contribution of the third frequency. Nevertheless, the observation noise of the IF-PPP2 model is slightly smaller than that of the IF-PPP0 and IF-PPP1 models.

Current precise satellite clock corrections are conventionally associated with the P1/P2 code IF combination. To use and remain compatible with precise clocks, the IFB should be considered for all three triple-frequency PPP models [16]. Satellite DCBs can be corrected in advance by using multi-GNSS experiment (MGEX) DCB products. For the receiver, the code biases caused by the IFB parameter should be introduced in the IF-PPP1 model to compensate for the different effects on different frequency bands, and for the IF-PPP2 model, it can be absorbed by the receiver clock parameter and thus cause system bias in precise time transfer.

With regard to the estimates, the receiver coordinates and wet troposphere delays can be considered model-independent. The other estimates, such as receiver clock, IFB, and ambiguities, are associated with a particular model or combination. As the IFB is much stable, it can be removed by the parameter estimation and prediction with long time datasets or by a priori calibration. In this study, the IFB is estimated as a constant by adding the priori value constraint which predicted by last epoch. In addition, the estimable clocks of IF-PPP0 and IF-PPP1 are referred to as the P1/P2 IF clock (${\mathrm{dt}}_{12}$), whereas the estimated receiver clock of IF-PPP2 is the combined effect of (${\mathrm{dt}}_{12}$) and receiver hardware delays (shown in Equation (13)). The ambiguity estimates are also associated with different frequencies.

(13) $$dt_{123}=dt_{12}+\left[\left(e_{2}-\beta_{12}\right)\cdot DCB_{r,12}+e_{3}\cdot DCB_{r,13}\right]$$

## 4. Validations

To validate the feasibility and effectiveness of the proposed model, an experiment was performed at the National Time Service Center (NTSC), Chinese Academy of Sciences, in LinTong in ShaanXi Province. The datasets were collected from day 299 to day 301 in 2017. The time link is a short baseline (several meters) between the NTSA and NTSC stations, which are connected to a common external time frequency from the NTSC laboratory. The sample rate was 30 s, and it can track the BDS B1, B2, and B3 signals. The receiver type was Trimble NetR9 and antenna type was RNG80971.00.

Coordinates with precision better than 1 cm are provided by the PPP week solutions, and they can be used for evaluating the accuracy [8].

**Figure 1:**
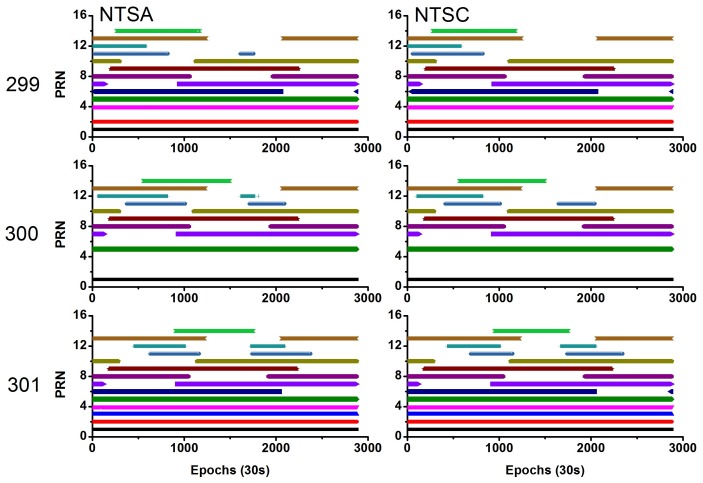
PRN (pseudo random noise code) of the visible BeiDou Navigation Satellite System (BDS) satellites during the experiment (**left** side represents NTSA station, and the **right** side represents National Time Service Center (NTSC) station).

**Figure 2:**
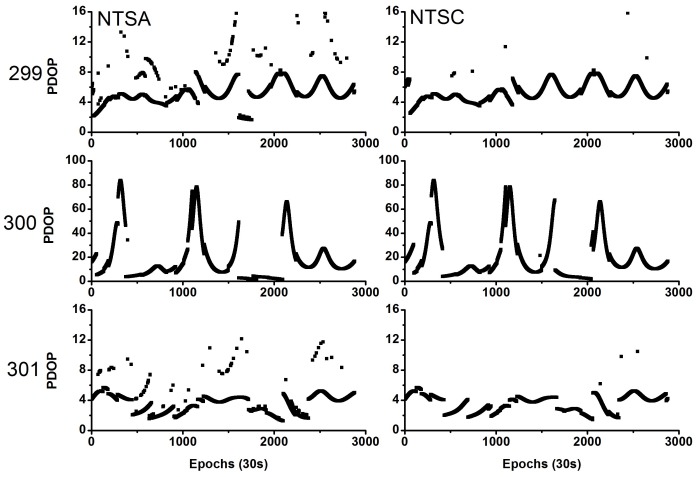
Position dilution of precision values for NTSA (**left**) and NTSC (**right**) stations.

Figure 1 shows the observed and useable BDS satellites for the two stations during the experimental period. The average number of visible satellites was (9, 9), (6.5, 6.5), and (9, 9) for the NTSA and NTSC stations on the three days, respectively. In addition, Figure 2 shows the position dilution of precision (PDOP) values for these stations. The average PDOP values were (5.23, 5.03), (17.89, 17.78), and (3.59, 3.58) for the NTSA and NTSC stations on these three days, respectively. On day 300, some of the observed satellites were deleted as their orbit and clock products had low precision, thereby resulting in fewer useable satellites and larger PDOP values on this day.

### 4.1. Analysis of Clock Time Series

**Figure 3:**
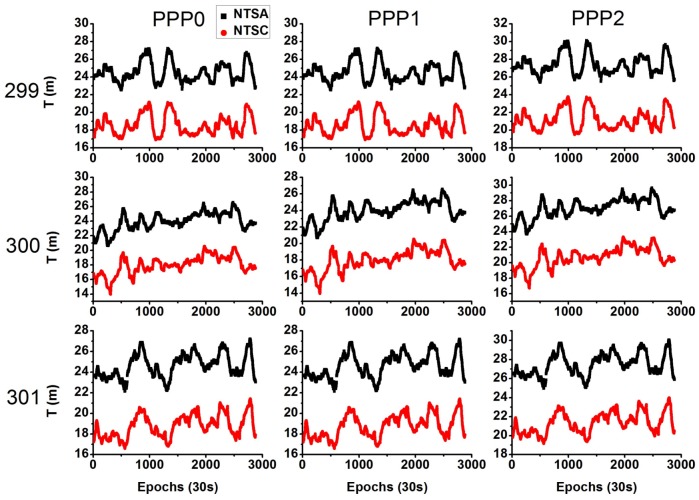
Comparison of clock time series between different solution models (The vertical offset between the NTSA and NTSC clock traces is a system bias related to the hardware delay).

Figure 3 shows the raw clock time series. It can be seen that the estimated receiver clock has the same variation for different models each day; however, the variation is also different on different days. In addition, as the two receivers are connected to a common external time frequency, the clock time series of the two stations also have the same variation (mean STD of their difference is 0.005 m) except for a system bias related to the hardware delay.

### 4.2. Analysis of Inter Frequency Bias

**Figure 4:**
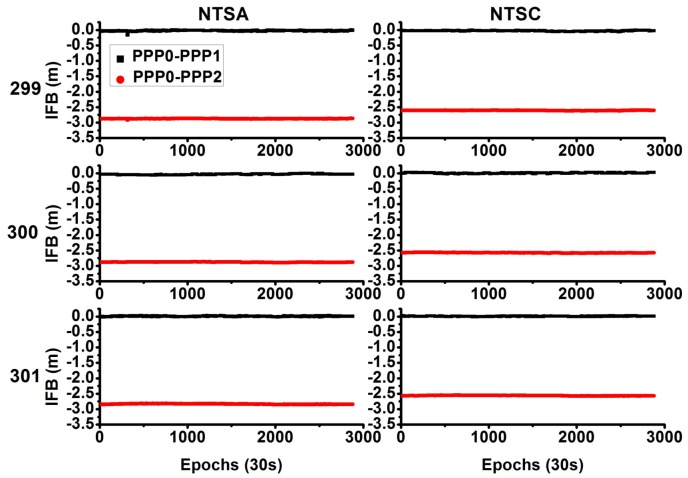
Comparison of inter frequency bias between different solution models (**left** side represents NTSA station, and **right** side represents NTSC station).

As discussed before, for the PPP1 model, the IFB has to be estimated, and for the PPP2 model, the estimated receiver clock includes the absorbed IFB. Figure 4 shows the estimated and recovered IFBs. It can be concluded that these biases are very stable, and the mean standard deviation (STD) is ~0.005 m; thus, it can be estimated as a constant in one day. As the IFB is receiver-independent, the IFB value is different for different stations. In addition, the IFB is also considerably stable for different days; therefore, we can use the predicted IFB value as a constraint for the parameter estimation. The difference between the PPP0-PPP1 and PPP0-PPP2 is a system bias related to the hardware delay.

### 4.3. Analysis of Time Differences

**Figure 5:**
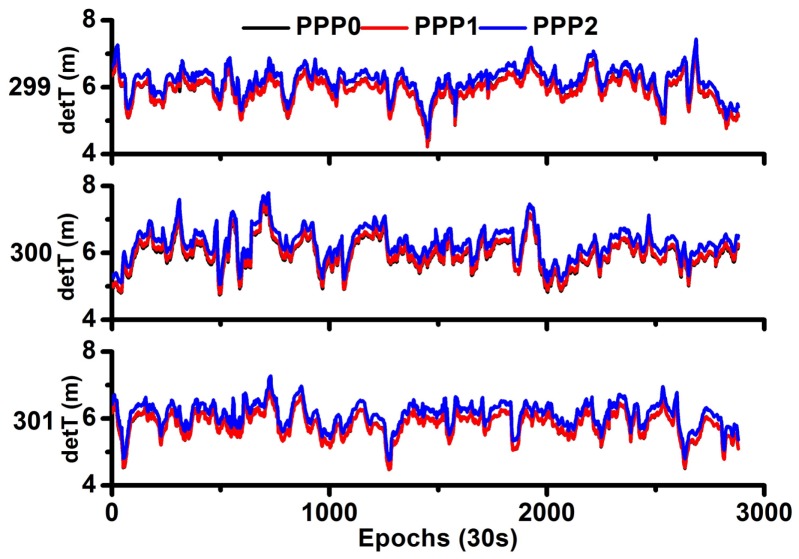
Comparison of time difference between different models.

The time difference is the bridge for precise time transfer. Figure 5 shows the time difference of the two stations. As they use the same external time frequency, the time difference reflects the hardware delay. It can be seen that the time difference is nearly the same except for the small system bias between the different models. As discussed before, the estimable clocks of IF-PPP0 and IF-PPP1 are referred to as the P1/P2 IF clock (${\mathrm{dt}}_{12}$), whereas the estimated receiver clock of IF-PPP2 is the combined effect of (${\mathrm{dt}}_{12}$) and receiver hardware delays. Thus, the time difference of two stations by the PPP0 and PPP1 models is the same, and the curves in Figure 5 are overlapped.

### 4.4. Analysis of System Bias

**Figure 6:**
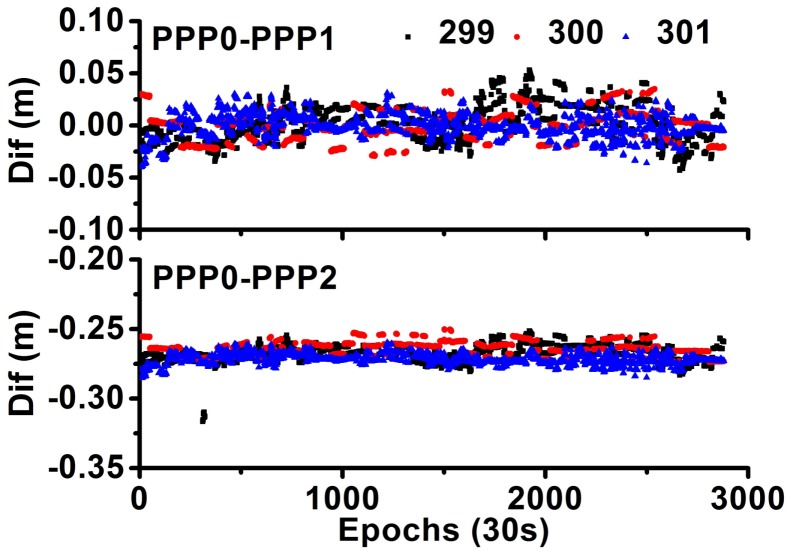
Comparison of system bias between different models.

Figure 6 shows the system biases; they are very stable for different days. In general, the system bias between PPP0 and PPP1 should be zero, as the PPP1 model estimates the IFB. As Figure 6 shows, for the PPP0 and PPP1 models, the bias of the time difference is considerably small, with mean values of (0.0025, −0.0005, −0.0010) m and STD of (0.019, 0.015, 0.011) m for these three days. For the PPP2 model, as the IFBs are absorbed by the receiver clock, the bias represents the difference of the hardware delay between different stations and different frequencies. As Figure 6 shows, for the PPP0 and PPP2 models, the bias of the time difference is very large, with mean values of (−0.2673, −0.2638, −0.2718) m and STD of (0.006, 0.005, 0.004) m for these three days. As Table 1 shows, the PPP2 model has the smallest noise compared to the PPP0 and PPP1 models; thus, the STD of the PPP2 model is also much smaller than that of the PPP1 model.

### 4.5. Analysis of Allan Deviation

**Figure 7:**
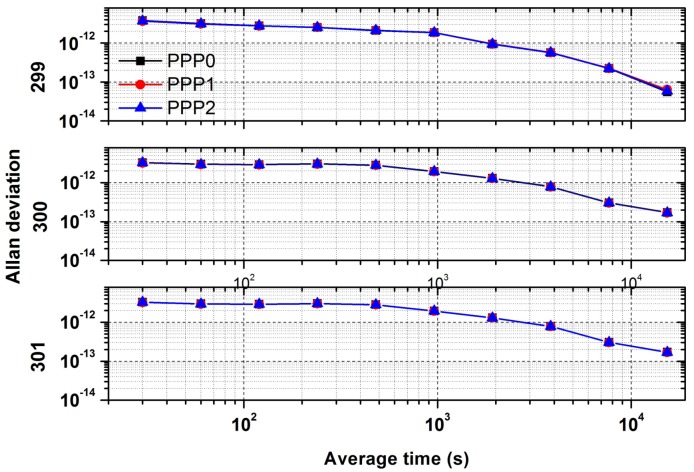
Comparison of Allan deviation between different models.

Figure 7 shows the Allan deviation values at different time intervals of the three models for precise time transfer, which is used to compare the performance of the three schemes, especially for the case of short term. The value of Allan Deviation is determined by one day data which contains 2880 data points and over a span of 86,400 s. As seen in this figure, the frequency stability is the same for the three different models. In addition, as the receivers are connected to a common external time frequency from the NTSC laboratory, for the three continuous days, the Allan deviation also has the same variation, and the stability of 10,000 s is better than 5 × 10^−13^.

### 4.6. Analysis of Positioning Error

**Figure 8:**
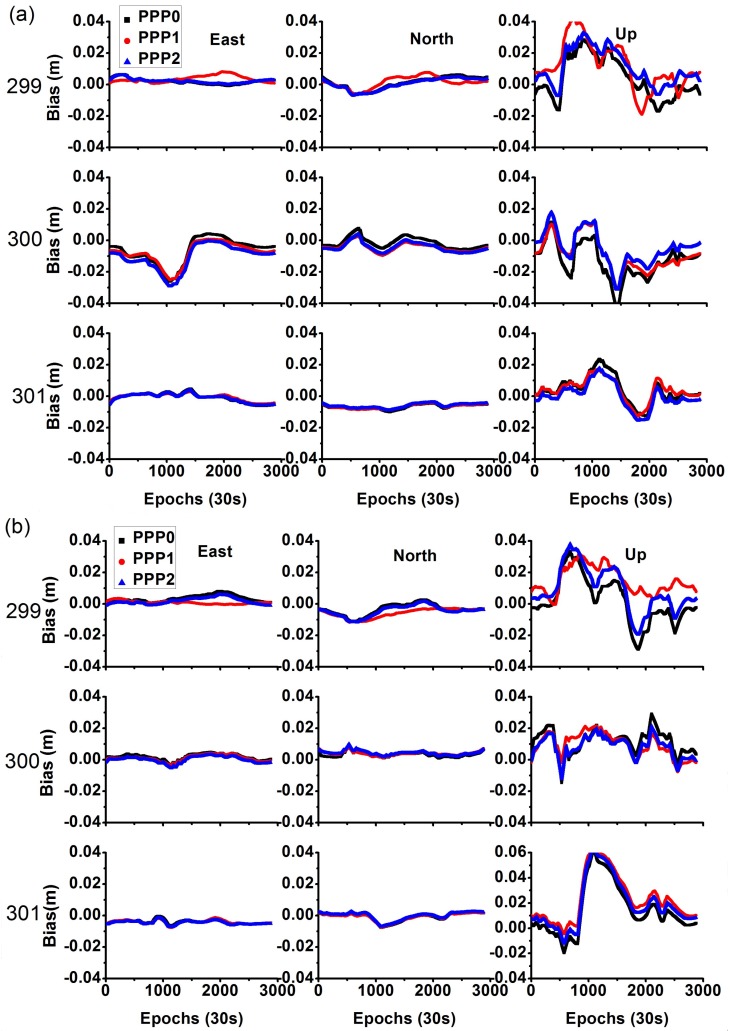
Comparison of position error between different models ((**a**) represents NTSA station, and (**b**) represents NTSC station).

For these time transfer models, the station coordinates are estimated as constants. Figure 8 shows the time series of the estimated coordinate errors. NTSA station showed a large fluctuation on day 300 owing to bad data quality. These results indicate that the positioning error is not significantly different between the different models, and the average STD is (1.5, 2.1, 7.3) mm in the north, east, and vertical components, respectively. Actually, for precise time transfer, the coordinates can be constrained with the prior values to enhance the parameter estimation.

### 4.7. Analysis of Troposphere

**Figure 9:**
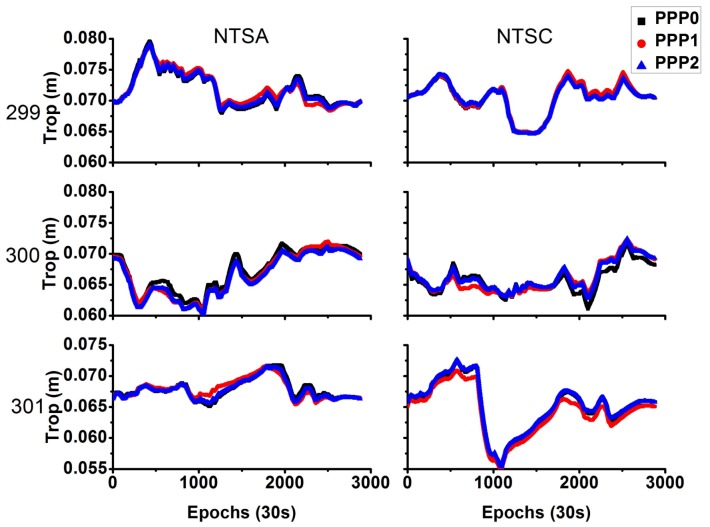
Comparison of residual tropospheric delay difference between different models (**left** side represents NTSA station, and **right** side represents NTSC station).

For these time transfer models, the residual tropospheric delay is estimated as a piecewise constant every hour. Figure 9 shows the time series of the estimated residual tropospheric delay. The three different models did not show a significant difference in the estimated tropospheric delays, with maximal difference of ~1 mm.

### 4.8. Analysis of Long Baseline Datasets

In order to better evaluate the performance of these time transfer models, other datasets were selected for the results analysis. The datasets were collected from day 302 to day 307 in 2017, the sample rate was 30 s, and it can track the BDS B1, B2, and B3 signals. The time link is a long baseline (1936.90 km) between the International GNSS Service (IGS) stations CEDU (Location: 133.8° E, 30.1° S, Receiver: SEPT POLARX5, Antenna: AOAD/M_T) and KAT1 (Location: 132.2° E, 13.6° S, Receiver: SEPT POLARX5, Antenna: AOAD/M_T).

**Figure 10:**
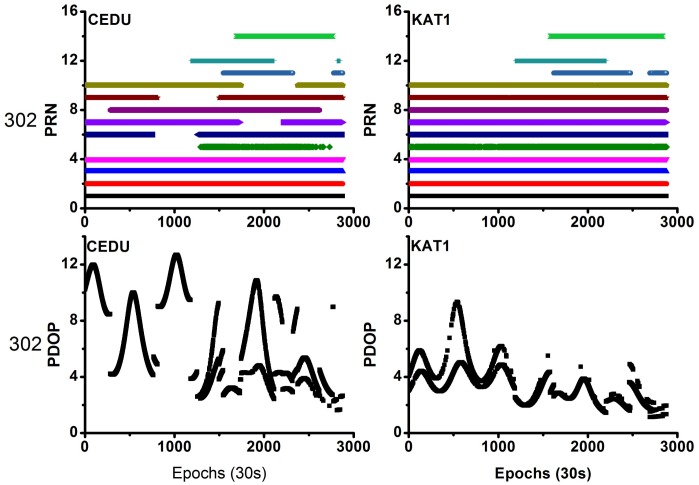
PRN and position dilution of precision (PDOP) values on day 302 (**left** side represents CEDU station, and the **right** side represents KAT1 station).

Figure 10 shows the observed BDS satellites and PDOP values for the two stations on day 302. The average number of visible satellites was (9.2, 10.9), and the average PDOP values were (6.21, 3.30) for the CEDU and KAT1 stations on day 302, respectively. The satellite conditions and PDOP values of the other five days are nearly the same with day 302.

**Figure 11:**
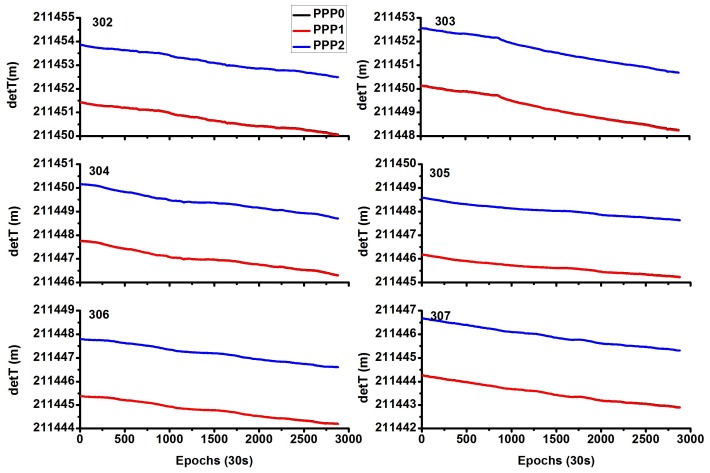
Comparison of time difference between different models by stations CEDU (**left** side) and KAT1 (**right** side).

Figure 11 shows the time difference of the stations CEDU and KAT1. It can be seen that the time difference of two stations by the PPP0 and PPP1 models is nearly the same, and the curves (black and red) in Figure 11 are overlapped. The time difference is also with the same tendency except for the system bias between the PPP0/PPP1 and PP2 models for this long baseline stations, these system bias is also caused by the hardware delay.

**Figure 12:**
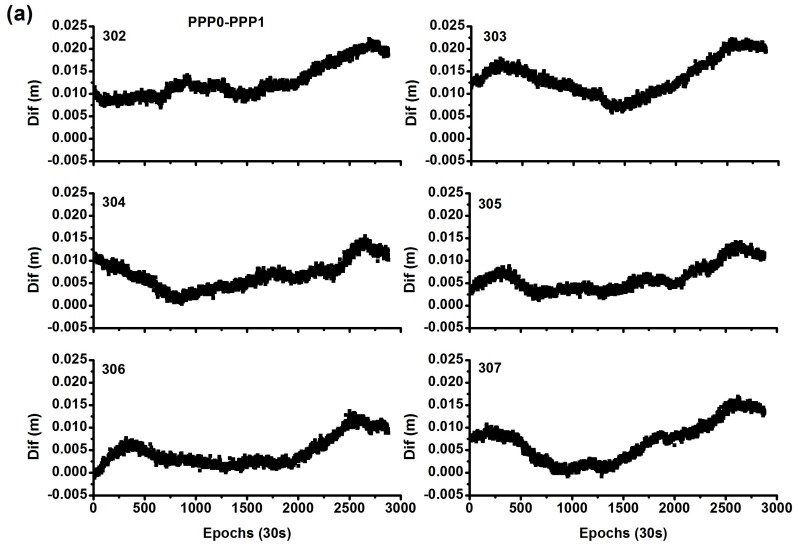

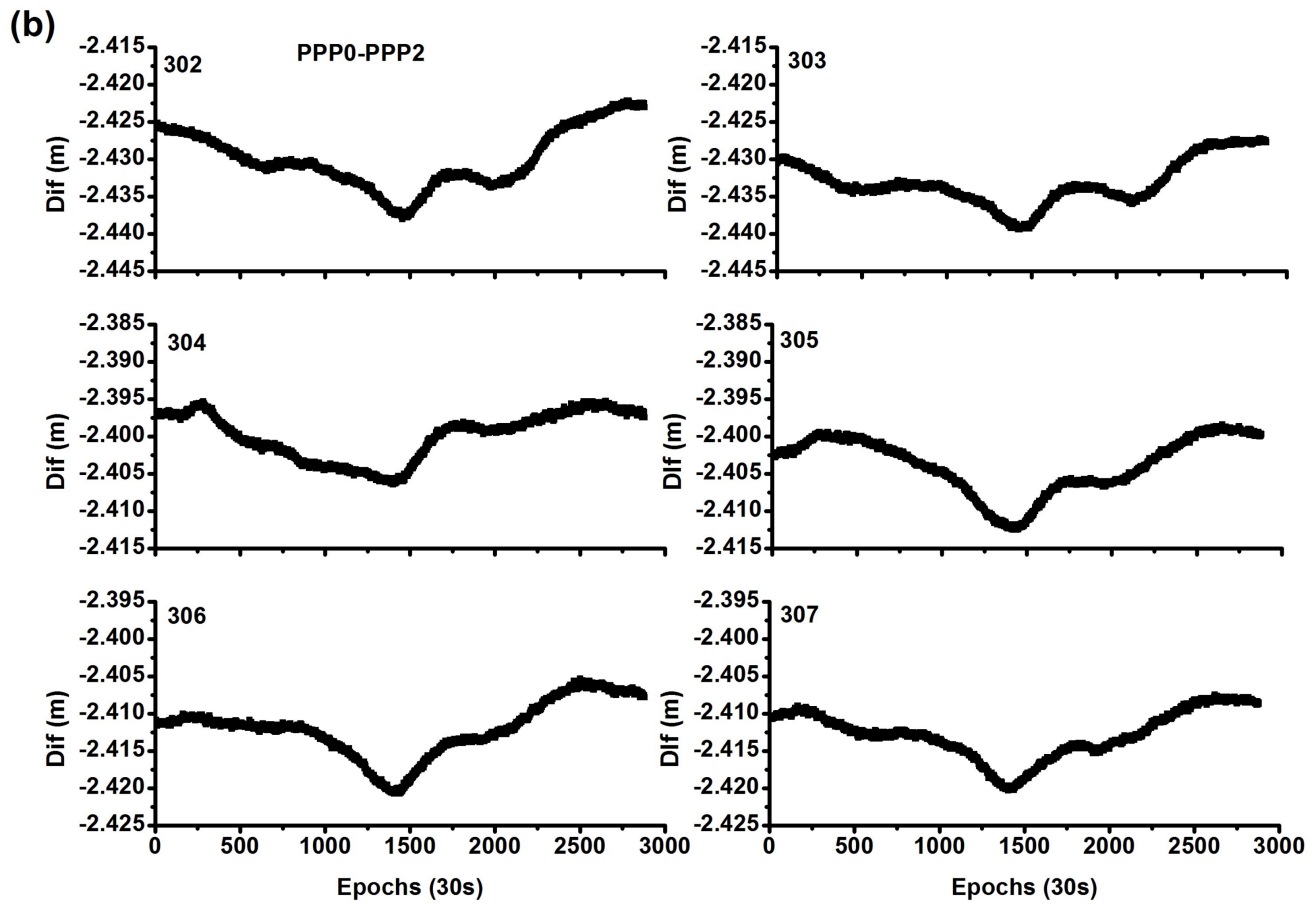
Comparison of system bias between different models by stations CEDU and KAT1 ((**a**) represents “PPP0-PPP1”, (**b**) represents “PPP0-PPP2”).

Figure 12 shows the system biases; they are very stable for different days. As Figure 12 shows, for the PPP0 and PPP1 models, the bias of the time difference is considerably small, with mean values of (0.012, 0.013 0.007, 0.007, 0.005, 0.007) m and STD of (0.004, 0.004, 0.003, 0.003, 0.003, 0.004) m for these six days. And for the PPP0 and PPP2 models, the bias of the time difference is very large, with mean values of (−2.430, −2.433, −2.400, −2.403, −2.412, −2.413) m and STD of (0.004, 0.003, 0.003, 0.004, 0.004, 0.003) m for these six days.

**Figure 13:**
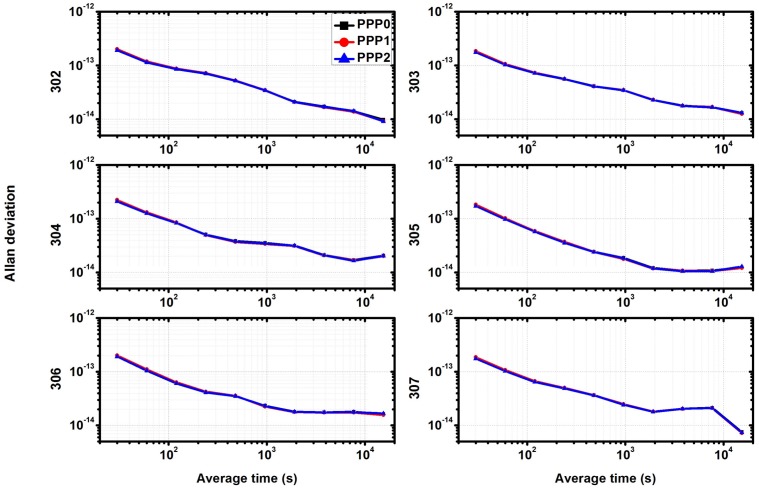
Comparison of Allan deviation between different models by stations CEDU (**left** side) and KAT1 (**right** side).

Figure 13 shows the Allan deviation values. As seen in this figure, the frequency stability is the same for the three different models, thus the curves are overlapped. In addition, for the six continuous days, the Allan deviation also has the same variation, and the stability of 10,000 s is better than 5 × 10^−1^^4^.

## 5. Conclusions and Discussion

In this study, we proposed two models for precise time transfer by using BDS triple-frequency signals: the IF-PPP1 model with two dual-frequency combinations and the IF-PPP2 model with a single triple-frequency combination. Datasets with a short baseline and a long baseline were used to assess the feasibility and effectiveness of the proposed models. From these validations and analysis, the following conclusions were obtained:

(1) Both the PPP1 and PPP2 models can be used for precise time transfer by using BDS triple-frequency signals, and the accuracy and stability of time transfer is nearly the same in both cases. The coordinates and troposphere delay estimates also have the same precision.
(2) The IFB caused by the hardware delay should be considered in the PPP1 model. It is stable each day and can be estimated as a constant, and it will be absorbed by the receiver clock in the PPP2 model that cannot be separated from the raw receiver clock. Therefore, there is a time difference for two stations between the PPP0 and PPP2 models with a stable system bias.
(3) It is important to note that all the PPP models used the zero-differenced observations, all the errors are precisely corrected or estimated, thus the time transfer results by PPP technology are not influenced by the location of the users.

In this study, all PPP models used IF combinations. Recently, the zero-differenced and zero-combined PPP models have been widely studied; these models have smaller noise, can directly obtain the ionosphere total electron content (TEC) and DCB products, and can correct ionosphere delay errors. In the future, uncombined PPP models for precise time transfer with BDS triple-frequency signals will be studied. In addition, more extensive experiment with longer run-times and a range of baselines should be done to better evaluate the performance of these time transfer models.

## Figures and Tables

**Table 1:** Comparisons of dual- and triple-frequency precise point positioning (PPP) models.

Model	Obs.	e_1_	e_2_	e_3_	Ion.	Noise	Sat. DCB	Rec. DCB
PPP0	B1/B2	2.487	−1.487	0	0	2.90	0	0
PPP1	B1/B2	2.487	−1.487	0	0	2.90	0	0
	B1/B3	2.944	0	−1.944	0	3.53	${\mathrm{DCB}}_{\mathrm{PPP}1}^{S}$	estimated IFB
PPP2	B1/B2/B3	2.566	−1.299	−0.337	0	2.86	${\mathrm{DCB}}_{\mathrm{PPP}2}^{S}$	absorbed by clock
